# Parasites and Pathogens of the Honeybee (*Apis mellifera*) and Their Influence on Inter-Colonial Transmission

**DOI:** 10.1371/journal.pone.0140337

**Published:** 2015-10-09

**Authors:** Nadège Forfert, Myrsini E. Natsopoulou, Eva Frey, Peter Rosenkranz, Robert J. Paxton, Robin F. A. Moritz

**Affiliations:** 1 Institute of Biology, Martin-Luther-University Halle-Wittenberg, Halle (Saale), Germany; 2 University of Hohenheim, Apicultural State Institute, Stuttgart,Germany; 3 Department of Zoology and Entomology, University of Pretoria, Pretoria, South Africa; University of North Carolina, Greensboro, UNITED STATES

## Abstract

Pathogens and parasites may facilitate their transmission by manipulating host behavior. Honeybee pathogens and pests need to be transferred from one colony to another if they are to maintain themselves in a host population. Inter-colony transmission occurs typically through honeybee workers not returning to their home colony but entering a foreign colony (“drifting”). Pathogens might enhance drifting to enhance transmission to new colonies. We here report on the effects infection by ten honeybee viruses and *Nosema* spp., and *Varroa* mite infestation on honeybee drifting. Genotyping of workers collected from colonies allowed us to identify genuine drifted workers as well as source colonies sending out drifters in addition to sink colonies accepting them. We then used network analysis to determine patterns of drifting. Distance between colonies in the apiary was the major factor explaining 79% of drifting. None of the tested viruses or *Nosema* spp. were associated with the frequency of drifting. Only colony infestation with *Varroa* was associated with significantly enhanced drifting. More specifically, colonies with high *Varroa* infestation had a significantly enhanced acceptance of drifters, although they did not send out more drifting workers. Since *Varroa-*infested colonies show an enhanced attraction of drifting workers, and not only those infected with *Varroa* and its associated pathogens, infestation by *Varroa* may also facilitate the uptake of other pests and parasites.

## Introduction

Host-parasite coevolutionary arms races are characterized by adaptations and counter-adaptations, including adaptive behavioral changes, to increase parasite fitness [1]. For example, parasites can alter host behavior to favor their transmission by increasing their contact rate with uninfected, susceptible hosts [1]. In contrast, hosts can modify their own behavior to limit the spread of parasites [1], resulting in classical host parasite arms races that are expected to be particularly dynamic in social insects [2]. Once inside the colony, transmission is typically swift among nestmates whereas transmission between colonies is often a major problem for the parasite.

The Western honeybee (*Apis mellifera* L.) represents an excellent model for studying infection-induced behavioral changes in pathogen and host because it can easily be experimentally manipulated [2]. In addition, the important ecosystem service of pollination that it provides through the pollination of crops and wild flora is nowadays threaten by the decline in the numbers of managed colonies in Europe and North America [3,4]. Drivers of colony losses are thought to include parasites, pathogens, pesticides and their interactions [5–8]. Thus, gaining information on factors influencing honeybee parasite and pathogen transmission may contribute to an understanding of colony losses.

Horizontal inter-colony transmission of honeybee pathogens can take various routes, including: (1) contact between infected individuals or infectious materials during robbing (robbing bees invade another colony to steal food resources); (2) contact between infected and uninfected individuals from different colonies during foraging; (3) contact with infectious material from the environment; and (4) ‘drifting’ of an infected bee from its own to another colony [9]. A suite of other factors in addition to pathogens is known to enhance drifting. In particular, artificially high colony density at the apiary, similarity in hive design, and apiary layout profoundly affect the drifting of bees [10–12]. Thus, beekeepers have developed various techniques to reduce orientation errors of honeybees and facilitate their return to their home colony (e.g. spacing hives and using different hive entrance colors [12–20].

Since orientation is essential for the successful return of foraging social insects to their colony, any interference in orientation and learning skills will enhance drifting [21–23]. Indeed parasites (*e*.*g*. *Varroa destructor* and *Nosema ceranae*) have been shown to interfere with the homing ability of infested workers [21–23]. However, an impaired homing ability alone is insufficient to address the potentially enhanced drifting of workers into other colonies, as workers not returning to the colony might simply die in the environment. Thus the acceptance of infected workers into foreign colonies needs to be confirmed in order to show the actual, field-realistic impact of drifting on transmission.

To address this question, we here use an experimental design that not only allows us to determine the effects of *Nosema*, *Varroa* and *Varroa* associated viruses on drifting behavior of workers, but also to identify colonies serving as sources and hosts of drifted workers. The specific aim of this study was to determine the extent to which *Varroa* associated viruses, *Varroa* or *Nosema* enhance their own intra-colonial transmission by favoring drifting behavior of their honeybee hosts.

## Materials and Methods

### Experimental apiaries

Two apiaries with 14 colonies each in Kenzingen (K) (48°11′30″N 7°46′6″E) and Simonswald (S) (48°6′1″N 8°3′21″E) in SW Germany (total N = 28 colonies) served as experimental sites. In both apiaries, colonies were placed in a line of 18m. We enhanced the probably of drifting by using hives with similar shape and color and flight entrance orientation in the same direction. These conditions may not be representative of all apiary conditions, since many beekeepers provide hive markings to decrease drifting [14,15,17–19], but we needed a large sample size of drifted bees for conducting the experiment. Since the local orientation of workers is driven by local landmarks surrounding hive and the shape and color of a hive are more important for drifting than the linear distance between two hives [24], we present results from our analyses of the number of hives between colonies (hive sequence distance) rather than the linear distance between a pair of hives in our statistical analyses of drifting behavior. Biologically it seems more meaningful to use hive sequence distance because the linear distance would generate a false sense of precision less relevant to bee behavior. This was subsequently also reflected by a higher correlation between drifting and hive position rather than drifting and linear distance (see results). We note, however, that the apiary layout, with colonies distributed linearly, meant that ‘hive sequence distance’ and linear distance between a pair of colonies were themselves closely correlated.

In order to test for the impact of *Varroa* and its associated viruses on drifting, we used acaricide treatment to establish two groups of colonies, one group with a high and the other group with a low level of *Varroa* infestation. Initially, all colonies were treated before the experiment with two acaricides: CheckMite ® (active ingredient: 1.36 mg coumaphos; Bayer HealthCare AG, Leverkusen, Germany) and Bayvarol® (active ingredient: 4.0 mg flumethrin 90%; Bayer HealthCare AG) using one strip of each acaricide per hive box with brood to rid them of *Varroa* mites. On the 26^th^ of July 2011, we stopped the ChekMite and the Bayvarol treatment in one half of the colonies per site but retained treatment for the other half of the colonies until December 2011. Hence, at each site there were two groups with seven colonies each, one with low *Varroa* mite infestation (*i*.*e*. with continued acaricide treatment) and another group of colonies with high *Varroa* mite infestation (*i*.*e*. with discontinued acaricide treatment). Comparing the two groups allowed us to test if *Varroa* infestation had any impact on inter-colony drifting and the spread of pathogens.

### Sampling and genotyping

We aimed to collect an unbiased worker sample of both non-drifted and drifted workers from each colony, which we could genotype to differentiate between the two, drifted and non-drifted. Since workers can only drift once they fly and engage in tasks outside the hive, we sampled and genotyped old workers returning from foraging flights at the flight entrance. Only those passing guard bees and directly entering the hive on a straight flight path were sampled. We thereby avoided sampling foreign bees that might not have been accepted by the guard workers at the flight entrance and also workers on orientation flights that typically hover in front of the hive. Twelve (±1 s.e.) workers per colony (N = 328) were sampled on 26^th^ September 2011 as described. At the same time, a piece of sealed brood (5x5cm) containing 12 pupae was cut from the comb of each colony, freeze killed in dry ice, and transferred to the laboratory, where it was stored at -80°C until genotyping. Using pupae allowed us to determine the colony’s natal bee genotypes. It also allowed us to determine the colony of origin of drifted bees.

Individual pupae were genotyped using two sets of tightly linked microsatellite loci on chromosomes 13 and 16 (Table 1), which allowed us to infer the queen genotypes of all sampled colonies. We used sets of linked loci to enhance the resolution of the markers [25]. DNA was extracted with a standard solvent extraction protocol (phenol/chloroform) and precipitated with ethanol. Extracted DNA was amplified by multiplex PCRs containing 10 ng DNA in 1μl DNA dilution buffer (Qiagen), 400 pM of each primer, 1.25x reaction buffer (Sigma), 200 μM of each dNTP, 1U of *Taq*-polymerase and HPLC water to a final volume of 10μl. The temperature profile for the PCR was as follows: 5 min denaturation at 95°C, 35 cycles of 30 sec each for denaturation (95°C), annealing T_m_ (see Table 1) and extension (72°C), followed by a final step of 5 min at 72°C. The amplified products were resolved in an automated DNA capillary sequencer (MegaBACE 1000, GE Healthcare Europe GmbH, Freiburg, Germany), including an internal size standard (ET-Rox 400, GE Healthcare Europe GmbH, Freiburg, Germany). Fragment sizes were analyzed using MegaBACE Fragment Profiler Version 1.2.

**Table 1:** Microsatellite markers for genotyping and detection of drifters.

Name	Size	Dye	T_m_	Forward primer (5'-3')	Reverse primer (5'-3')	Chromosome
AC006	157	TET	54	GATCGTGGAAACCGCGAC	CACGGCCTCGTAACGGTC	16
A003	154	HEX	54	CGCCTGTGACGACGATGC	CAGCCGGCACATCTCCATC	16
AG005C	131	FAM	54	GGAGAACGTTGAACGTCGC	TCGCGCACAATCTTCACC	16
AT121	183	FAM	54	AACCGAGCCGAGTCTGGAATC	CGTCCCTCGATTCGTTTTTCTC	16
HB-C16-05	97	FAM	54	ATTTTATGCGCGTTTCGTA	CATGGCTCCTCCATTAAATC	16
HB-C16-03	113	HEX	54	CAAACAACTTCTCGAAAACA	CCGAAGAGAATAAATGGTAGA	16
6560	155	HEX	54	AATTCCAGCCGTCGGTCC	GAAAGTATCCAATATTTTCGCACG	16
K1628	120	HEX	54	GCTCGATTAAAGTCACGATTCC	AGAATCCACGCGAGCAATC	16
SV240	265	TET	55	CGTGCGCCCTTTTTGTCAC	CGGGACGGTTGATGATGAAG	13
HB004	198	HEX	55	CAAACAAACCGTGTGGATGT	ACTGCGAGGAAAAAGGAAGT	13
HB010	133	TET	55	CCGATTTAACCCTCCGATTA	GCGTGACGTTCAAGAAGAAT	13
HB017	131	HEX	52	TACGACCCATAACACGCAAT	GTTCGTGCCACCTTCTATTC	13
HB015	129	FAM	52	CGGTCGAGAGATGGTTGTAA	GTCATCCACTTTTCCCTTCA	13
HB005	221	TET	52	CGTTTCTCTACCCTCGAACA	ATCTGCCGAAAAGACTCTCA	13

### Detection of drifted workers

The DNA of the workers (N = 328) sampled at the flight entrance of each colony was extracted from a hind leg using a Chelex protocol [26]. The same genotyping procedures as for the brood were used. Natal and drifted workers were identified by comparing their genotypes with those of the colony’s queen, which was inferred from that of her diploid offspring (pupae). When an adult worker shared one of the queen’s alleles at both marker sets, it was considered to be the offspring of that queen. When an adult worker carried alleles different to those of the queen of that colony, it was considered to be a drifter. Its genotype was then compared to those of other queens of the apiary to find the matching one. In the few cases in which a drifter’s genotype matched with several queen genotypes (*i*.*e*. similar allele combinations at all loci of the two linkage groups), the genotypes of the siring males [27] of the respective queens were also used to infer the natal colony of that worker. Individuals with an allele combination that could not be assigned to any colony in the apiary were classified as drifters from “unknown” mother colonies.

### Pathogen detection

We focused on a suite of common RNA viruses and the two microsporidian gut pathogens: *Nosema ceranae* and *Nosema apis*, as parasites that potentially impact drifting behavior. Total RNA was extracted with an RNeasy Mini Kit (Qiagen) using a Qiacube robot (Qiagen) from the same adult workers (N = 328) that had been sampled at the flight entrance and genotyped. The presence of 10 viruses and 2 *Nosema* spp. was determined in individual bees by employing Multiple Ligation-dependent Probe Amplification (MLPA) and RT-PCR respectively. MLPA was performed as described [28] using the probes designed for detecting six targets of the positive single strand RNA viruses: (i) Chronic Bee Paralysis Virus (CBPV), (ii) Deformed Wing Virus (DWV)/Varroa Destructor Virus-1 (VDV-1)/Kakugo Virus (KV), (iii) Acute Bee Paralysis Virus (ABPV)/Israeli Acute Paralysis Virus (IAPV)/ Kashmir Bee Virus (KBV), (iv) Black Queen Cell Virus (BQCV), (v) Slow Bee Paralysis Virus (SBPV), and (vi) Sacbrood Bee Virus (SBV), with β-actine (housekeeping gene) as a control for RNA extraction and PCR. *Nosema* spp. detection was done by RT-PCR on total cDNA synthesized with M-MLV Revertase (Promega) according to the manufacturer’s instructions and using 800 ng total RNA. Species-specific primers were used as described in vanEngelsdorp et al. (2009) [4] but with a modified annealing temperature of 54°C. Amplicons were electrophoretically separated and visualised in a QIAxcel instrument (Qiagen) and scored positive or negative using a threshold of 0.1 relative fluorescence units [29,30].

In addition to virus and *Nosema* spp. detection, we determined the *Varroa* infestation rate in a sample of ca. 150 worker bees per colony at the beginning of September, at the end of September and in mid-October [31]. We used the mean *Varroa* infestation determined over those three days of sampling in statistical analyses.

### Network analysis

We used a network analysis to explore the flow of drifters between colonies. Each apiary was considered a separate network in which colonies were represented by “nodes” that were connected by “links”, referring to the number of drifters found in a non-natal colony [32]. Since any dispersal of workers was directional, with colonies receiving (sink colonies) and colonies sending drifters (source colonies), we generated a directional network in which we could differentiate between the *outdegree centrality* (high in a source colony) and the *indegree centrality* (high in a sink colony) as centrality indices for every colony [33]. Hence *outdegree* refers to the number of drifters sent from a given source colony, while *indegree* refers to the number of drifters received by a sink colony. These measures quantify how much a colony is central within the apiary in terms of sending and receiving drifters respectively. For example, *indegree centrality* may be driven by the scrutiny of guard bees screening incoming worker bees, or some other colony-based mechanism, but not necessarily by a trait of the individual drifting worker.

### Statistical analyses

The *Varroa* mite infestation rate between the acaricide treated and untreated colonies was compared using a Mann-Whitney U-test. We used Chi^2^ tests to compare the proportion of drifters between both sites. To assess the relationship between drifting and the distance between sink and source colony in an apiary, we used the number of colonies between source and sink as the inter-hive distance; to do so, we conducted a Spearman's rank correlation between the number of drifted bees and the inter-hive distance; analyses were repeated using linear distance between two hives in place of inter-hive distance. The proportions of drifters between treated and untreated source and sink colonies were compared using a Chi^2^ test of homogeneity (all expected numbers >5). The level of infection by pathogens (viruses and *Nosema* spp.) between individual native and drifting bees of the same natal colonies was compared using a Fisher exact test (theoretical frequencies <5). Probabilities were adjusted for multiple comparisons using a Bonferroni *post-hoc* correction. Since workers were sampled after returning from a flight, the determination of individual mite infestation at take-off or during the flight could not be determined since mites may be lost during flight.

**Fig 1:**
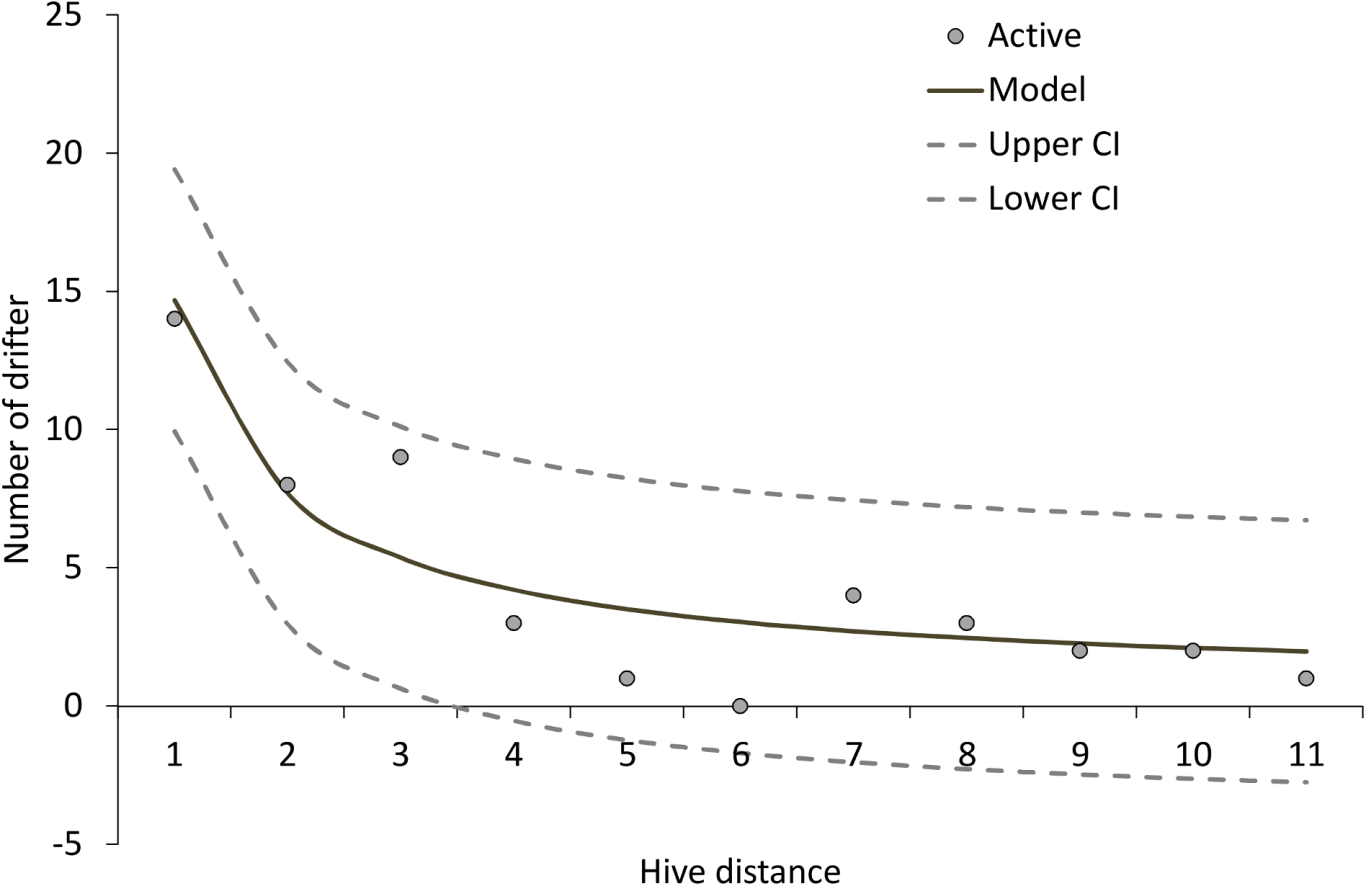
Number of bees drifting from neighbouring colonies (N = 47). The distance of “one” means that the bees came from the neighbouring colony, “two” from the colony next to the neighbouring colony and so on. The equation for the relationship is: y = a + b/x where a = 0.716 and b = 13.96.

The *outdegree centrality (OC)* and *indegree centrality (IC)* for each colony at each site were computed using the software Visone 2.8. Since we wanted to discriminate between drifting that was a consequence of inter-hive distance and drifting that was a consequence of pathogens, we first determined the relationship between drifting and inter-hive distance using non-linear regression (Fig 1). Based on this function, we developed a network of expected drifting based on inter-hive distance alone to determine both *OC* and *IC* for each colony of each site. We then compared these expected *OC* and *IC* values with those of the network resulting from the observed drifting between colonies. We used the difference between the observed and expected values of *OC* and *IC* (Δ*OC* and Δ*IC*) to evaluate the effect of pathogens alone on drifting. Only parasites and pathogens that could potentially have affected drifting were included in this analysis. These comprised viruses and *Nosema* spp., which differed in prevalence between non-drifting and drifting bees, as well as *Varroa*, which differed in infestation across colonies through our experimental acaricide treatment. Since each site represents a unique network, we ran separate network analyses, one for each site. We used Mann-Whitney U-tests to assess if colony infection or *Varroa* infestation was associated with drifting. This approach allows for the comparison of both apiary networks in a single analysis. Statistical comparisons were conducted using R software version 3.1.1. [34].

### Ethics statement

Both localities were private and permission was given by local beekeepers to enter them and sample from colonies. In Germany, specific permission for setting up an apiary is not required if the owner of the location agrees to it. The only requirement is a document stating that colonies are free of American foulbrood, which had been issued by veterinarian authorities for our experimental colonies. Also specific permission is not required for the sampling of honeybees for the diagnosis and analysis of pests and bee diseases (*i*.*e*. *Varroa*, *Nosema*, viruses). Our experiments were exclusively performed with honeybees, insects that are not under particular protection or belong to endangered or protected species.

## Results

### Colony *Varroa* mite infestation

As expected from our experimental design, bees in colonies that were treated against *Varroa* were significantly less infested (0.17% ± 1.21 s.e. *Varroa* mites per 150 workers) than in untreated ones (5.17% ± 0.06 s.e. *Varroa* mites per 150 workers) (U-test, p-value = 10^−6^). The ‘untreated’ colonies (*high Varroa*) had a 30 times higher *Varroa* mite infestation than the continuously treated ones (*low Varroa*).

### Detection of drifted workers

From the 328 adult worker honeybees genotyped and screened for pathogens, 253 workers were native bees in their natal colony and 75 drifters (with an average of 17 ± 4% per colony). Significantly more drifters were identified at the Simonswald apiary (55 out of 167 workers) than at Kenzingen (20 out of 161 workers) (Chi^2^ = 18.41, df = 1, p < 0.001). Twenty-eight drifted workers did not have a genotype corresponding to any of the queens and fathering drones at an apiary, thus their colony of origin could not be assigned.

### Factors affecting drifting

#### Inter-hive distance

We first determined the relationship between inter-hive distance at the apiary and drifting of workers (N = 47). We found a significant negative correlation between drifting and inter-hive distance (Spearman rank correlation: rho = -0.79; p = 0.006). The relationship between drifting and linear distance between a pair of hives was also significantly negative (rho = -0.55, p = 0.022), but weaker than for inter-hive distance so we used inter-hive distance in further analyses. We estimated the effect of inter-hive distance on drifting using a non-linear regression (Fig 1) as a best fit model (R^2^ = 0.85). We therefore corrected for hive position in subsequent analyses when testing for the impact of parasites and pathogens on drifting.

### Individual infection

**Fig 2:**
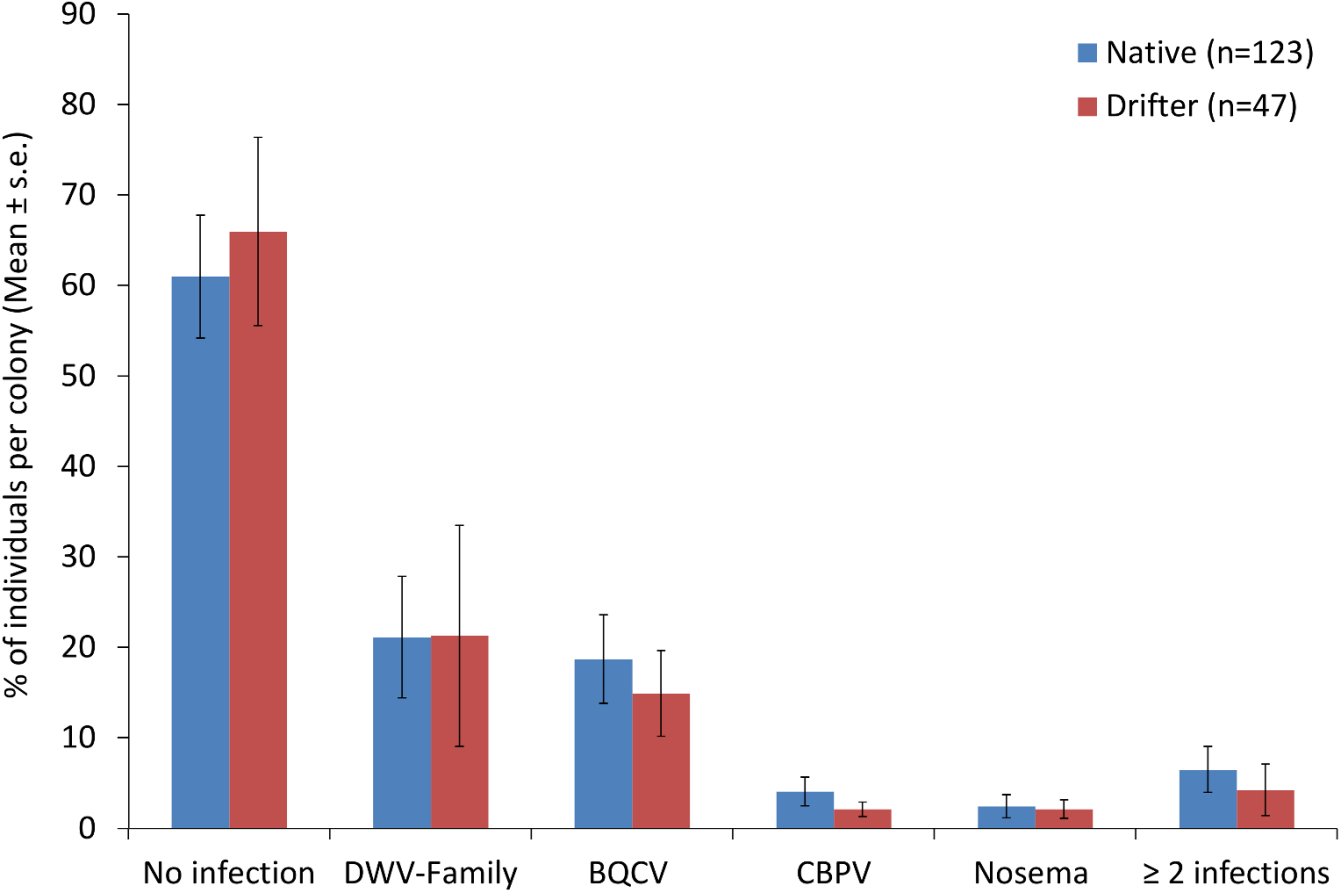
Infection of drifter and native bees. To assess the effect of pathogens on drifting behavior, viruses and *Nosema* spp. infections were compared between the drifters and the native bees of the sink colonies (N = 14 colonies).

We tested if drifters were more infected by the various tested pathogens (viruses and *Nosema* spp.) than non-drifters of a given source colony. From our 28 colonies, 14 were a source of drifters. Within the 14 colonies, we compared pathogen prevalence in a total of 123 native workers and 47 workers that had drifted out of these colonies into others within the same apiary. Among the six virus families, three were not detected in our samples: ABPV/IAPV/KBV, SBPV and SBV. In total, 34% of the drifters and 39% of the natives carried at least one pathogen. Our samples were a snap-shot; native workers might also drift later in their lives. There was no significant difference in the proportion of infected drifters versus infected native bees, either for all pathogens combined (Fisher’s exact tests: p-value = 0.13) or for individual pathogens (Fisher’s exact tests: DWV-Family: p-value = 0.60; BQCV: p-value = 0.33; CBPV: p-value = 0.17; *N*. *ceranae*: p-value = 0.60, see Fig 2). Natives were significantly more infected by ≥2 pathogens than drifters (Fisher’s exact test: p-value = 0.026). However, after Bonferroni correction for multiple comparisons, differences were not significant (p-value ≥ 0.05). The minimum detectable prevalence in a sample size of n = 123 for natives and n = 47 for drifters is thus 1 out of 47 (ca. 6%; binomial error at p<0.05). This may lead to a slight inaccuracy in the estimation of pathogen prevalence and the effect of pathogens on drifting.

#### *Varroa* infestation

The majority of the drifters (68%) with an assigned colony of origin (N = 47) came from colonies with high mite infestation and significantly less (32%) from colonies with low mite infestation (Chi^2^ = 5.72, df = 1, p = 0.017). In addition, significantly more drifters were sampled from colonies with high *Varroa* mite infestation (Chi^2^ = 17.38, df = 1, p = 0.00003). However, drifting was strongly affected by hive position (Fig 1), which confounds the effect of *Varroa* alone on drifting. We therefore used a network approach to correct for inter-hive distance and extract the residual impact of *Varroa* infestation on drifting from the data set.

### Network analysis

**Fig 3:**
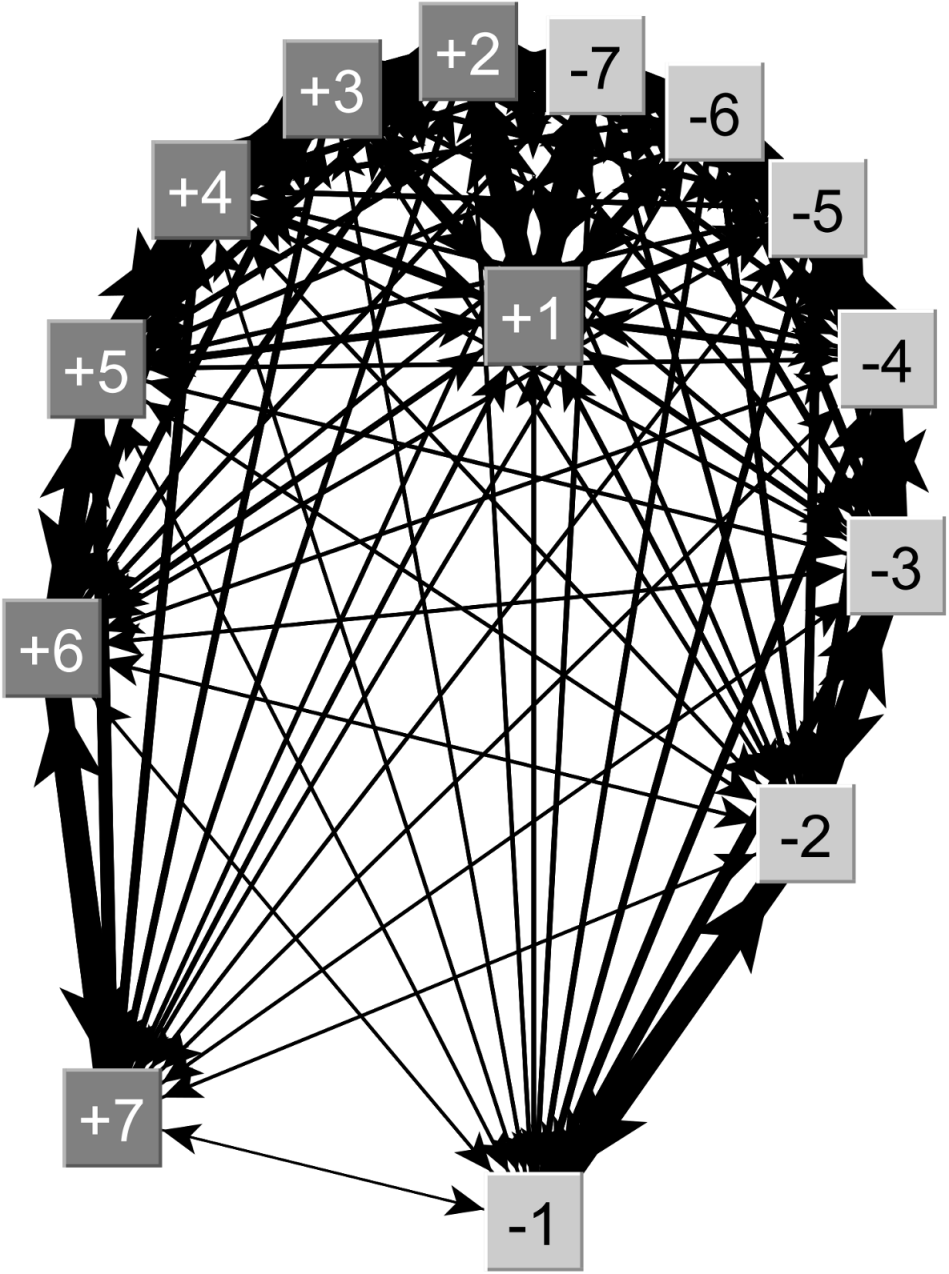
Network map of colonies from Simonswald on accepting drifters according to their *indegree centrality*. Here we represent the observed *indegree centrality* based on the actual number of drifters. Each square represents a colony (“+”high *Varroa*; “-” low *Varroa*) whilst the number refers to the colony position at the apiary, with +1 and -1 being the two central hives and +7 and -7 those at the two ends of the row. The arrows represent the flow of drifters and their width is proportional to the number of drifters (from 1 to 4) going from one colony to another. Only Simonswald network is represented since more drifters were found in this site than in Kenzingen, which illustrates better our results.

As only *Varroa* infestation–yet none of the screened pathogens–yielded a significant association with drifting, we focused the network analysis on *Varroa* with respect to *outdegree* (*OC*) and *indegree centrality* (*IC*). After correcting for inter-hive distance, we found that colonies with high *Varroa* infestation accepted significantly more drifters (Δ*IC* = 2.76) than those with low *Varroa* infestation (Δ*IC* = -2.76; U-test p = 0.036) (Fig 3 (A) and 3 (B)). The level of *Varroa* infestation of source colonies had a negative but non-significant effect on *OC* (*high Varroa*: Δ*OC* = 2.15, *low Varroa*: Δ*OC* = -2.15, U-test: p>0.05); in other words, there was a non-significant trend for *high Varroa* colonies to send out more drifters than *low Varroa* colonies.

## Discussion

For a honeybee parasite, a crucial point in its life cycle is transmission between colonies [2]. A general strategy to enhance transmission is to manipulate host behavior so as to be vectored from one host to another [1]. We here studied the impact of *Varroa* mites, *Varroa*-associated viruses and *Nosema* on one of the major routes for honeybee pathogens to be transmitted between colonies, honeybee drifting [9]. Only *Varroa* mite enhanced drifting, with infested colony accepting more drifter than colonies with low *Varroa* infestation, while neither *Varroa*-associated viruses nor *Nosema* seem to have an impact on drifting behavior.

We conducted our experiment in an apicultural setting, where colony density is several orders of magnitude higher than in wild honeybee populations [35–37]. In such a context, drifting of infected bees has been shown to be a major transmission pathway [10–13]. Though the rate of drifting between colonies in natural populations is not known, it is suggested to be substantially lower than in managed apiaries [10,11]. As a consequence, it has been proposed that the lower level of infection (e.g. infestation by *Varroa* mites) of scattered and isolated feral honeybee colonies than those at apiaries may be driven by diminished drifting and inter-colony pathogen transmission in the latter [10,38,39].

Most beekeepers adopt techniques to minimize the impact of drifting on the health of their colonies [12,13,15,16,20]. We did not use hive markings enhancing visual cues for bee orientation as we aimed to generate many drifters to obtain satisfactory sample sizes. Yet it has been shown that specific hive colors and a more spaced apiary layout can only partly overcome the effects of the artificially high colony density at large commercial apiaries. The apiary as such is inevitably a prime facilitator of inter-colony drift [16,40].

We found significant differences in the frequency of drifted workers between our two experimental sites. However, since we had only two sites we can only speculate on potential reasons, which may include resource abundance and local landmarks. Yet it was not our aim to look at general environmental effects on drifting but rather to dissect those factors that are relevant at the within-apiary level.

The position of the hive in the apiary was most important and explained 79% of the variance in observed drifting, which primarily occurred among neighboring colonies. Hence efforts to prevent drifting by improved apiary layout or by providing extra landmark (e.g. unique entrance marks) may not just prevent drifting and the transport of *Varroa* in late summer but may reduce pathogen transmission among colonies throughout the season [11,31]. Packing colonies in tight rows in the apiary will lead to enhanced transmission.

None of the viruses found in our study (BQCV, the DWV-family and CBPV) or *Nosema* spp. showed significant associations with drifting. Since the *Varroa* treatment successfully allowed us to create two different groups of colonies with low and high *Varroa* infestation, we could more clearly infer the effect of this parasite on drifting (see S1 Table). Only infestation with *V*. *destructor* contributed significantly to drifting. However, this was not at the level of the individual worker. Since we only screened for drifted workers that had been accepted into a sink colony, any mites on these bees at sampling were neither informative about its original source colony nor about the infestation of the worker bee when it had actually drifted. Hence, the effects of *Varroa* we measured were at the colony level rather than at the level of the individually drifting worker.

The network analyses allowed us to correct for hive position and extract the effects of *Varroa* mites on drifting in more detail. On the one hand, drifting of bees is driven by the individual behavior of workers coming from source colonies. This can either be due to impaired orientation of workers returning from flights, or altered behavior induced by the pathogen or pest to enhance its inter-colony transmission [2]. On the other hand drifting also requires the host colony accepting the foreign worker. The network analyses strongly indicated that any effect of *Varroa* on the individual drifting bee is less important (a reduced but non-significant *outdegree centrality* of the low *Varroa* colonies) but rather operates at the level of the colony when accepting drifted workers (sink colony: significantly higher *indegree centrality* in high *Varroa* colonies).

Enhanced acceptance of drifters may have been due to an impaired ability to scrutinize incoming foreign workers by guard bees. Recently, Annoscia et al. (2015) [41] showed that *Varroa* infestation during the pupal stage alters the in-hive behavior of ensuing adult honeybees, resulting in reduced activity and participation in hive duties. If this were also true for guarding behavior, the higher acceptance of drifters by *Varroa* infested colonies may be linked to reduced guarding efficiency of resident workers. Thus, in light of our results it seems that *Varroa* may affect the colony at a global level or at least the behaviour of its guarding bees to favor the transmission of new incoming *Varroa* mites. Indeed it might be adaptive for the mites to enhance their population in the colony to escape its rigid reproductive system of inbreeding. This extreme inbreeding can only be avoided if a host pupa is infected with more than a single mite lineage, as seen in the Asian honeybee *Apis cerana* [42].

We cannot completely exclude a direct effect of the acaricide treatment with coumaphos and flumethrin on adult worker bees in the *low Varroa* colonies. Since these compounds have been reported to impair olfactory learning and memory [43,44], one might expect such colonies to send out more drifters because of their reduced orientations skills. Moreover, one might expect guard bees in these colonies, which identify incoming workers based on learned odor cues, to be less efficient. However, our results show exactly the opposite, which suggests that acaricide treatment as such had little, if any, effect on drifting. It seems unreasonable to assume that acaricides could enhance the ability of guard workers in rejecting foreign drifters from drifting into the *low Varroa* colonies, since acaricides impair and not enhance olfactory learning [43,44]. Also, the acaricides we used to generate *low Varroa* colonies did not promote workers drifting out of the colony into others.

However, we cannot exclude that the acaricides had a repellent effect, reducing the drifting of workers from other colonies into low *Varroa* colonies. In spite of this caveat, we consider repellence unlikely because both acaricides have low volatility and rather operate by contact. If active as repellent compounds, they would probably require contact between native and foreign workers. However, from an applied perspective, even if they repel drifting workers, it would represent an added benefit to acaricide treatment by reducing horizontal transmission of pests and associated pathogens among colonies. In any case, it is advantageous to keep *Varroa* infestation low in late summer not only to prevent reaching the *Varroa* damage threshold [45] but also to reduce horizontal transmission of pests and associated pathogens among colonies.

Krajl and Fuchs (2006) [21] reported on *Varroa* mites impairing homing efficiency of foragers. They released infested foragers at some distance from the hive and noted that these took longer to return home than non-infested ones, or did not return at all. The authors interpreted their results as an adaptive behavior of the bees to remove parasites or pathogens from the colony. In a subsequent study, Kralj and Fuchs (2010) [22] investigated the effect of *Nosema* spp. on the flight behavior of forager bees using a similar experimental paradigm and found similar results. Also Wolf et al. (2014) [46] reported that *Nosema-*infected bees failed to return home. The increased homing failure of *Nosema*-inoculated bees was explained though energetic stress induced by the infection leading to bees running out of energy on their path back home. If *Varroa* infested or *Nosema* infected returning bees simply disappear in the environment and do not return at all to any colony, then drifting would not be enhanced.

Whereas these former studies [21,22,46] could not draw firm conclusions on drifting *per se* since only impaired flight or homing failure were assessed, our study directly assessed drifting events; we found that drifters did not show higher *Nosema* infection than native bees. Hence, although *Nosema* interferes with flight behavior, orientation and the ability of bees to return to their home colony, we found no evidence that *Nosema* increases drifting of infected bees.

*Varroa* is not only a bee parasite but also acts as vector for several honeybee pathogens. Hence any increased transmission through drifting workers induced by *Varroa* or its associated pathogens would not only be beneficial to the mite itself but also to the pathogens it carries. In spite of this theoretical selective advantage, we were not able to detect such increased drifting induced by any of the *Varroa-*associated pathogens in our data set. Only, *Varroa* infestation at the colony level elevated the drifting of foreign workers into that colony. Although *Varroa*-associated pathogens did not show a direct effect on drifting of workers, *Varroa* infested colonies are nevertheless likely to acquire more pathogens since their probability of receiving drifters and hence also pathogens was greater than in colonies with low *Varroa* infestation. At the same time, pathogens are easily spread from bee to bee within the colony via *Varroa* as a vector. In addition *Varroa* has been hypothesized to serve as a replicator for pathogens in the colony [47]. These colonies will therefore eventually serve as sources of pathogens for healthy colonies, eventually spreading pathogens into neighbouring colonies and across the apiary.

Apiary layout and density, by facilitating inter-colonial transmission, uncouples the trade-off between virulence and transmission typically seen among pathogens and parasites [48]. The apiary provides an ideal ground for highly virulent pathogens such as DVW because transmission is also by *Varroa* parasitism, reducing the ability of a colony to stem inter-colony transmission.

## Supporting Information

S1 Table Viruses and *Nosema* infection, *Varroa* infestation and proportion of honeybee drifters (*Apis mellifera*) detected in acaricides treated or untreated colonies in two different apiaries sites, Kenzingen (K) and Simonswald (S), Germany. At each apiary, seven colonies were treated against *Varroa* and seven were untreated. Honeybee foragers were sampled flying back to the hive after passing the guarding bees. Among them, individuals were identified as drifters. For some of them, their source colony could not be identified. The level of viruses and *Nosema* infections is based on the infection of the native foraging bees. *Varroa* infestation was determined from an independent sample of 150 in-hive bees.
(DOCX)
