# Supplementary material for: Parasites and Pathogens of the Honeybee (Apis mellifera) and Their Influence on Inter-Colonial Transmission
Source: PLoS One. 2015 Oct 9;10(10):e0140337. doi: 10.1371/journal.pone.0140337 (PMC4599887; doi:10.1371/journal.pone.0140337)
Supplement: S1 Table — At each apiary, seven colonies were treated against Varroa and seven were untreated. Honeybee foragers were sampled flying back to the hive after passing the guarding bees. Among them, individuals were identified as drifters. For some of them, their source colony could not be identified. The level of viruses and Nosema infections is based on the infection of the native foraging bees. Varroa infestation was determined from an independent sample of 150 in-hive bees. (DOCX) [file pone.0140337.s001.docx]

| Site | Colony | Treatment | Nr. sampled bee | Nr. drifter | Nr. drifter from unknown colony source | DWV-family prevalence (%) | BQCV prevalence (%) | CBPV prevalence (%) | *Nosema* spp. prevalence (%) | Rate of *Varroa* infestation (%) |
| --- | --- | --- | --- | --- | --- | --- | --- | --- | --- | --- |
| K | 1 | No | 10 | 0 | - | 80 | 40 | 0 | 10 | 14.40 |
| K | 2 | No | 10 | 0 | - | 30 | 10 | 0 | 0 | 10.20 |
| K | 3 | No | 10 | 0 | - | 10 | 10 | 0 | 0 | 2.77 |
| K | 4 | No | 18 | 8 | 1 | 20 | 20 | 0 | 0 | 9.23 |
| K | 5 | No | 17 | 4 | 2 | 38.46 | 23.08 | 0 | 0 | 7.03 |
| K | 6 | No | 10 | 0 | - | 30 | 50 | 0 | 10 | 11.70 |
| K | 7 | No | 11 | 0 | - | 0 | 36.36 | 9.09 | 0 | 4.77 |
| K | 8 | Yes | 16 | 6 | 0 | 50 | 50 | 0 | 10 | 0.27 |
| K | 9 | Yes | 11 | 2 | 2 | 0 | 22.22 | 0 | 0 | 0.40 |
| K | 10 | Yes | 9 | 0 | - | 11.11 | 55.55 | 0 | 0 | 0 |
| K | 11 | Yes | 9 | 0 | - | 0 | 33.33 | 0 | 0 | 0.60 |
| K | 12 | Yes | 10 | 0 | - | 10 | 20 | 0 | 0 | 0 |
| K | 13 | Yes | 10 | 0 | - | 0 | 30 | 0 | 0 | 0 |
| K | 14 | Yes | 10 | 0 | - | 0 | 20 | 0 | 0 | 0.40 |
| S | 15 | No | 17 | 14 | 9 | 0 | 0 | 0 | 0 | 0.60 |
| S | 16 | No | 11 | 2 | 1 | 0 | 33.33 | 0 | 0 | 2.37 |
| S | 17 | No | 15 | 8 | 2 | 42.86 | 28.57 | 0 | 14,29 | 1.033 |
| S | 18 | No | 14 | 5 | 3 | 44.44 | 22.22 | 0 | 0 | 2.67 |
| S | 19 | No | 8 | 3 | 0 | 20 | 0 | 0 | 0 | 1.87 |
| S | 20 | No | 17 | 9 | 3 | 0 | 0 | 12.5 | 0 | 3.10 |
| S | 21 | No | 10 | 4 | 2 | 0 | 0 | 16.67 | 0 | 0.63 |
| S | 22 | Yes | 10 | 0 |  | 0 | 0 | 0 | 10 | 0.13 |
| S | 23 | Yes | 15 | 5 | 0 | 20 | 10 | 10 | 0 | 0.20 |
| S | 24 | Yes | 15 | 2 | 2 | 15.38 | 0 | 7.69 | 7,69 | 0 |
| S | 25 | Yes | 12 | 1 | 0 | 9.09 | 0 | 0 | 0 | 0.43 |
| S | 26 | Yes | 7 | 2 | 1 | 60 | 20 | 0 | 0 | 0 |
| S | 27 | Yes | 6 | 0 | - | 16.67 | 16.67 | 0 | 0 | 0 |
| S | 28 | Yes | 10 | 0 | - | 10 | 0 | 0 | 0 | 0 |
